# The relative contribution of diurnal and nocturnal glucose exposures to HbA1c in type 1 diabetes males: a pooled analysis

**DOI:** 10.1007/s40200-022-01015-1

**Published:** 2022-03-31

**Authors:** Matthew D. Campbell, Daniel J. West, Lauren L. O’Mahoney, Sam Pearson, Noppadol Kietsiriroje, Mel Holmes, Ramzi A. Ajjan

**Affiliations:** 1 Faculty of Health Sciences and Wellbeing, University of Sunderland, Sunderland, SR1 3SD UK; 2 Leeds Institute for Cardiovascular and Metabolic Medicine, University of Leeds, Leeds, UK; 3 Human Nutrition Research Centre, Newcastle University, Newcastle, UK; 4 Population Health Science Institute, Faculty of Medical Science, Newcastle University, Newcastle, UK; 5 Diabetes Research Centre, Leicester General Hospital, University of Leicester, Leicester, UK; 6 Faculty of Medicine, Prince of Songkla University, Songkhla, Thailand

**Keywords:** CGM, Postprandial glucose, Glucose variability, HbA1c, Type 1 diabetes

## Abstract

**Purpose:**

The exact contribution of daily glucose exposure to HbA1c in people with type 1 diabetes (T1D) remains controversial. We examined the contribution of pre- and postprandial glycaemia, nocturnal and early-morning glycaemia, and glycaemic variability to HbA1c levels in T1D. In this analysis, we used clinical data, namely age, BMI and HbA1c, as well as glycaemic metrics (24-h glycaemia, postprandial, nocturnal, early-morning glycaemia, wake-up glucose, and glycaemic variability) obtained over a four-week period of continuous glucose monitoring (CGM) wear in thirty-two males with T1D.

**Methods:**

The trapezoid method was used estimate the incremental area under the glucose curve (iAUC) for 24-h, postprandial (3-h period following breakfast, lunch, and dinner, respectively), nocturnal (between 24:00–04:00 AM), and early-morning (2-h period 2-h prior to wake-up) glycaemia. Linear regression analysis was employed whereby CGM-derived glycaemic metrics were explanatory variables and HbA1c was the outcome.

**Results:**

Thirty-two T1D males (mean ± SD: age 29 ± 4 years; HbA1c 7.3 ± 0.9% [56 ± 13 mmol/mol]; BMI 25.80 ± 5.01 kg/m^2^) were included in this analysis. In linear models adjusted for age and BMI, HbA1c was associated with 24-h mean glucose (*r*^2^ = 0.735, *p* < 0.001), SD (*r*^2^ = 0.643, *p* = 0.039), and dinner iAUC (*r*^2^ = 0.711, *p* = 0.001). CGM-derived metrics and non-glycaemic factors explained 77% of the variance in HbA1c, in which postprandial glucose accounted for 32% of the variance explained. The single greatest contributor to HbA1c was dinner iAUC resulting in 0.6%-point (~7 mmol/mol) increase in HbA1c per SD increase in dinner iAUC.

**Conclusions:**

Using comprehensive CGM profiling, we show that postprandial glucose, specifically evening-time postprandial glucose, is the single largest contributing factor to HbA1c in T1D.

**Trial registration number:**

NCT02204839 (July 30th 2014); NCT02595658 (November 3rd 2015).

## Introduction

The impact of excessive glucose exposure on the development of long-term diabetes complications is well established [1–4] with studies showing unequivocally that improved long-term glucose control prevents and delays complications and reduces mortality [1, 5–9]. HbA1c is widely accepted as the hallmark measure of long-term glucose control, and this serves as the principal basis for treatment decisions aimed at reducing the risk of complications in people with type 1 diabetes (T1D). HbA1c reflects time-averaged mean blood glucose over a 8–12 week period and is derived from a composite of fasting and mealtime glucose responses [10], and impacted by glycaemic variability [11] as well as non-glycaemic parameters such as nutritional deficiencies, genetic factors, and personal characteristics including age and adiposity [12, 13]. However, the exact contribution of day-to-day glucose exposure to variation in HbA1c has not been fully established in T1D.

The advent of continuous glucose monitoring (CGM) technology, enables comprehensive glucose profiling for sustained periods of time under free living conditions, providing a unique opportunity to assess the individual contribution of discrete time intervals (e.g. mealtimes, overnight periods, and early mornings) as well as glycaemic variability to HbA1c. The ability to identify which aspects of every-day living contribute most to long-term glycaemic control, is important to our understanding of how treatment interventions or changes to daily self-management may improve long-term outcomes in T1D. In this current analysis, we aimed to compare the strength of associations across a range of CGM-derived glycaemic metrics with HbA1c levels in patients with T1D. Further, we estimated the variance in HbA1c explained by pre- and postprandial glycaemia, nocturnal glycaemia, glycaemic variability, and non-glycaemic factors.

## Methods

The present study consisted of the reanalysis of data from two previous RCTs (Clinical trial registration: clinicaltrials.gov NCT02204839; NCT02595658. Both RCTs received ethical approval from local National Health Service Research Ethics Committees (REC reference: 13/NE/0026; 14/NE/1183) and written informed consent was obtained from participants.

Detailed information regarding each study has been published previously [14, 15]. In the present analysis, we included participants meeting the following inclusion criteria: aged 18–50 years; classical presentation of T1D (including primary osmotic symptoms, weight loss, hyperglycaemia, ketosis, insulin initiation at diagnosis); diagnosed with T1D for a minimum of 5-years on enrolment; with stable HbA1c (less than a 0.2% change in HbA1c within a 12-month period); treated on a stable (>12-months) basal-bolus insulin regimen consisting of rapid-acting insulin analogues lispro or aspart and basal insulin glargine or determir delivered through multiple daily injections or continuous subcutaneous insulin infusion; and free of diabetes-related complications including background retinopathy.

In the present study, we used clinical data collected at baseline, namely age, BMI and HbA1c, as well as CGM-derived glycaemic metrics that were obtained over a four-week period. Glycaemia was captured under free-living conditions using a CGM system (Paradigm Veo, Medtronic Diabetes, Medtronic Minimed, USA) which records interstitial glucose concentrations continuously at 5-min intervals. CGM sensors (Enlite, Medtronic Diabetes, Medtronic Minimed, USA) were inserted into the subcutaneous tissue of the anterior-superior abdomen with insertion site replicated on subsequent sensor fitment. The site of sensor insertion was chosen to minimise the physiological time-lag between blood and interstitial concentrations. Measurements were performed for a minimum of 48-h at baseline, and repeated weekly for a total of 4 weeks. Only successful, uninterrupted (no gaps >15-min), profiles during pre-interventional periods were included in the analysis. So that initial calibration periods did not impact study results, we excluded the first two-hours of data following sensor placement. During CGM wear, participants were required to record a minimum 4-point self-monitoring blood glucose (SMBG) profile per 24-h period using a glucose testing meter (Glucomen Lx+, A. Menarini Diagnostics, UK) for calibration purposes; participants were required to capture SMBGs at least twice within a 12-h window including a reading immediately before bed, and immediately upon waking. In addition, dietary recording sheets were used to capture habitual diet patterns and establish meal-timing, as well as sleep, and wake times.

### Data processing

CGM-derived metrics included mean 24-h glucose, postprandial glucose (breakfast, lunch, and dinner), nocturnal glucose (captured between 24:00–04:00 AM), early-morning glucose (captured 2-h prior to waking), wake-up glucose, and glycaemic variability. We used the trapezoid method to estimate the incremental area under the glucose curve (iAUC) for the 24-h, postprandial, nocturnal, and early-morning periods. Glycaemic variability was assessed using 24-h data streams to calculate coefficient of variation (CV) as the primary measure of glycaemic variability, and standard deviation (SD) as a key secondary measure [16]. For each CGM-derived glycaemic metric, we averaged the values across each data capture from the four-week observation window to calculate a mean metric. Each mean CGM-derived glycaemic metric was then used as an explanatory variable with HbA1c at baseline as the outcome.

### Statistical analyses

Statistical analyses were performed in R version 4.0.0 (The R Foundation for Statistical Computing, Austria) and SPSS Statistics version 25 (IBM SPSS Statistics 25, IBM Corporation, USA). Descriptive characteristics of the study population are presented as mean ± SD; 95% confidence intervals (95%CI) and β coefficients are presented where relevant. To assess the association between CGM-derived glycaemic metrics a Pearson correlation coefficient matrix was applied. To estimate the associations of CGM-derived glycaemic metrics with HbA1c, we employed linear regression analysis without adjustment (model 1), and with adjustment for age and BMI (model 2). Prior to analyses, all CGM-derived metrics were normalised to facilitate direct comparisons of the strength of their respective associations with HbA1c. As such, the corresponding regression coefficients reflect the difference in HbA1c per 1 population SD (1-SD) difference for each CGM-derived metric. In unadjusted linear regression analyses we calculated the proportion of variance in HbA1c explained by CGM-derived metrics categorised as whole-day glycaemia (24-h mean glucose, 24-h mean iAUC), preprandial (nocturnal iAUC, early-morning iAUC, wake-up glucose), postprandial (breakfast iAUC, lunch iAUC, dinner iAUC), and glycaemic variability (CV, SD). To accommodate for correlations between CGM-derived metrics, we used their combined contribution to explained variance as a scaling factor to determine their individual relative contribution to explained variance in HbA1c [17]. In addition, we performed a sensitivity analysis, including only nocturnal iAUC, dinner iAUC, SD, age and BMI, as measures of pre- and postprandial glycaemia, glycaemic variability, and non-glycaemic factors, respectively. Statistical significance was set at *p* < 0.05 for all analyses.

## Results

### Clinical characteristics and CGM-derived glycaemic metrics

**Fig. 1:**
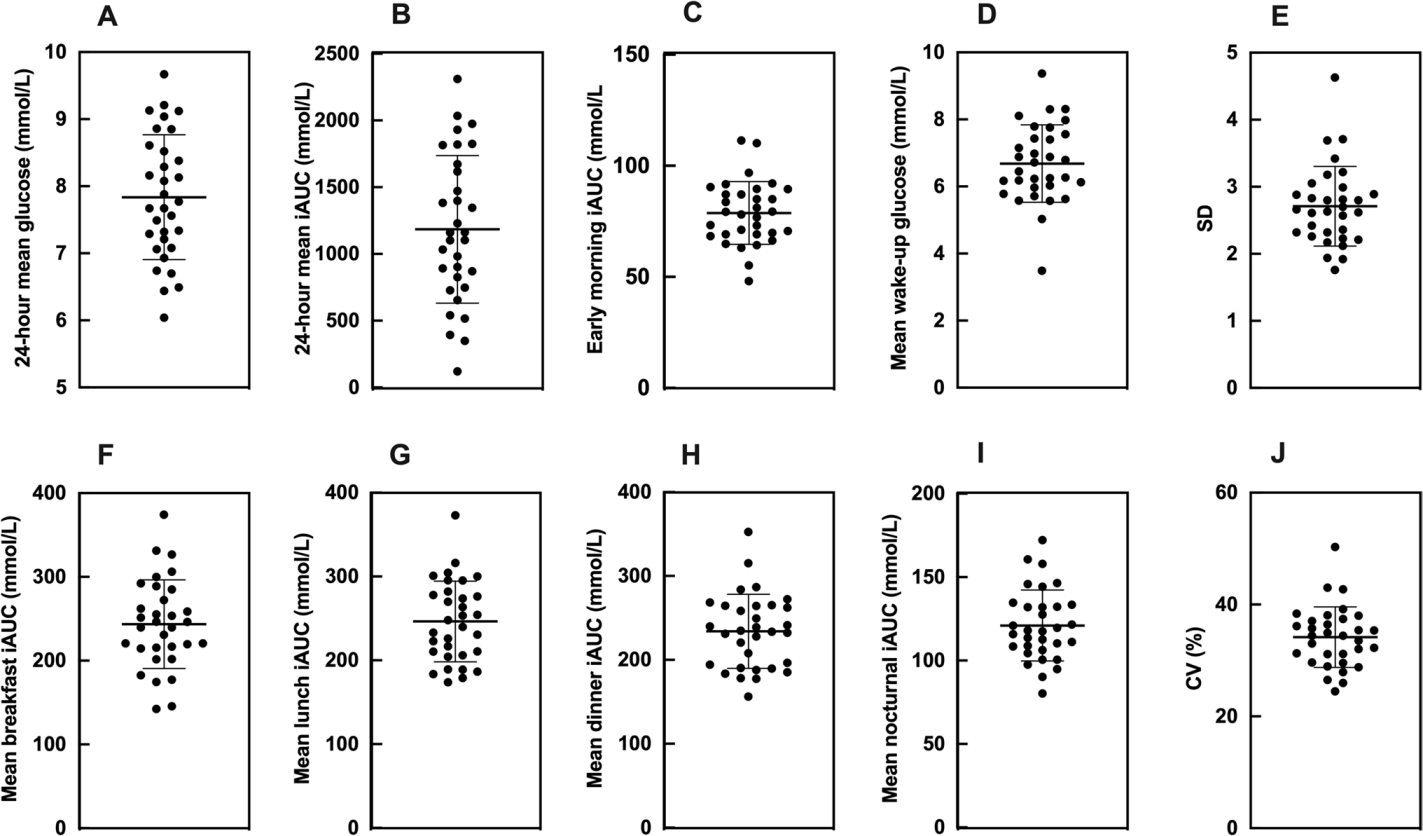
**A-J** Participant CGM-derived metrics. Breakfast, lunch, and dinner iAUC was calculated for 3-h after each meal. Early morning iAUC was calculated for a 2-h period prior to wake-up time. Nocturnal iAUC was calculated between 02:00–04:00 for all participants

**Fig. 2:**
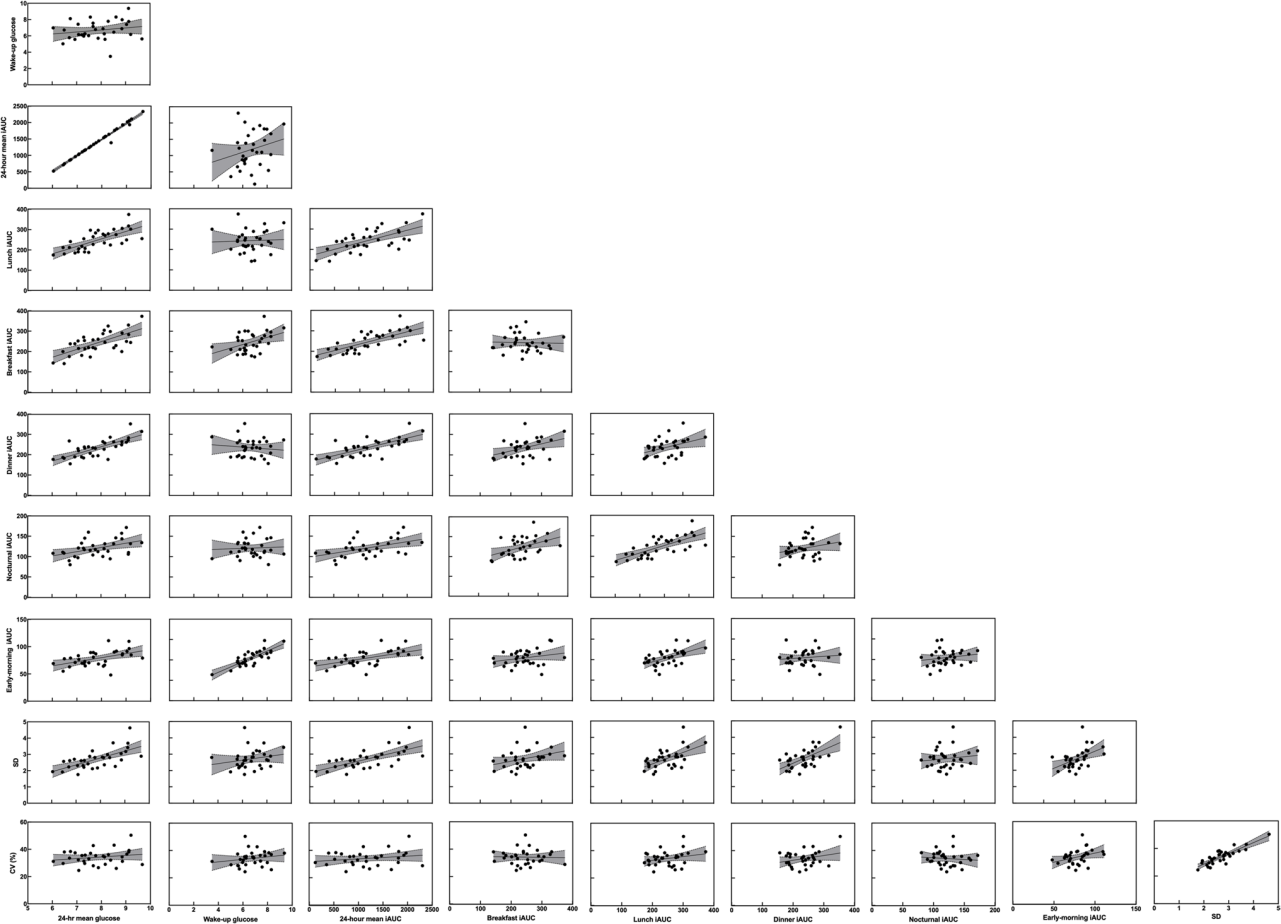
Pairwise scatter plots of the interrelationships between CGM-derived metrics with mean and 95%CI

Thirty-two T1D males were included in this study with a mean ± SD HbA1c of 7.3 ± 0.9% [56 ± 13 mmol/mol], a duration of T1D of 16 ± 10 years, a BMI of 25.80 ± 5.01 kg/m^2^, and an average age of 29 ± 4 years. The median number of days with valid CGM measurements in this study population was 8 [range: 4 to 16 days] over a minimum of a four-week period with at least one single uninterrupted 48-h period per week. Figure 1A-J shows the CGM-derived metrics for the cohort, and Fig. 2 shows pairwise scatter plots of the interrelationships between CGM-derived metrics. Mean 24-h glucose was highly correlated with breakfast iAUC (r = 0.672, *p* < 0.001), lunch iAUC (r = 0.707, *p* < 0.001), dinner iAUC (r = 0.740, *p* < 0.001), nocturnal iAUC (r = 0.454, *p* < 0.009), pre-breakfast iAUC (r = 0.494, *p* < 0.004), and SD (r = 0.667, *p* < 0.001), but not wake-up glucose (r = 0.202, *p* = 0.268), or CV% (r = 0.202, *p* = 0.267). The substitution of mean 24-h glucose for mean 24-h iAUC did not significantly alter associations.

### Relationships between CGM-derived glycaemic metrics and HbA1c

**Fig. 3:**
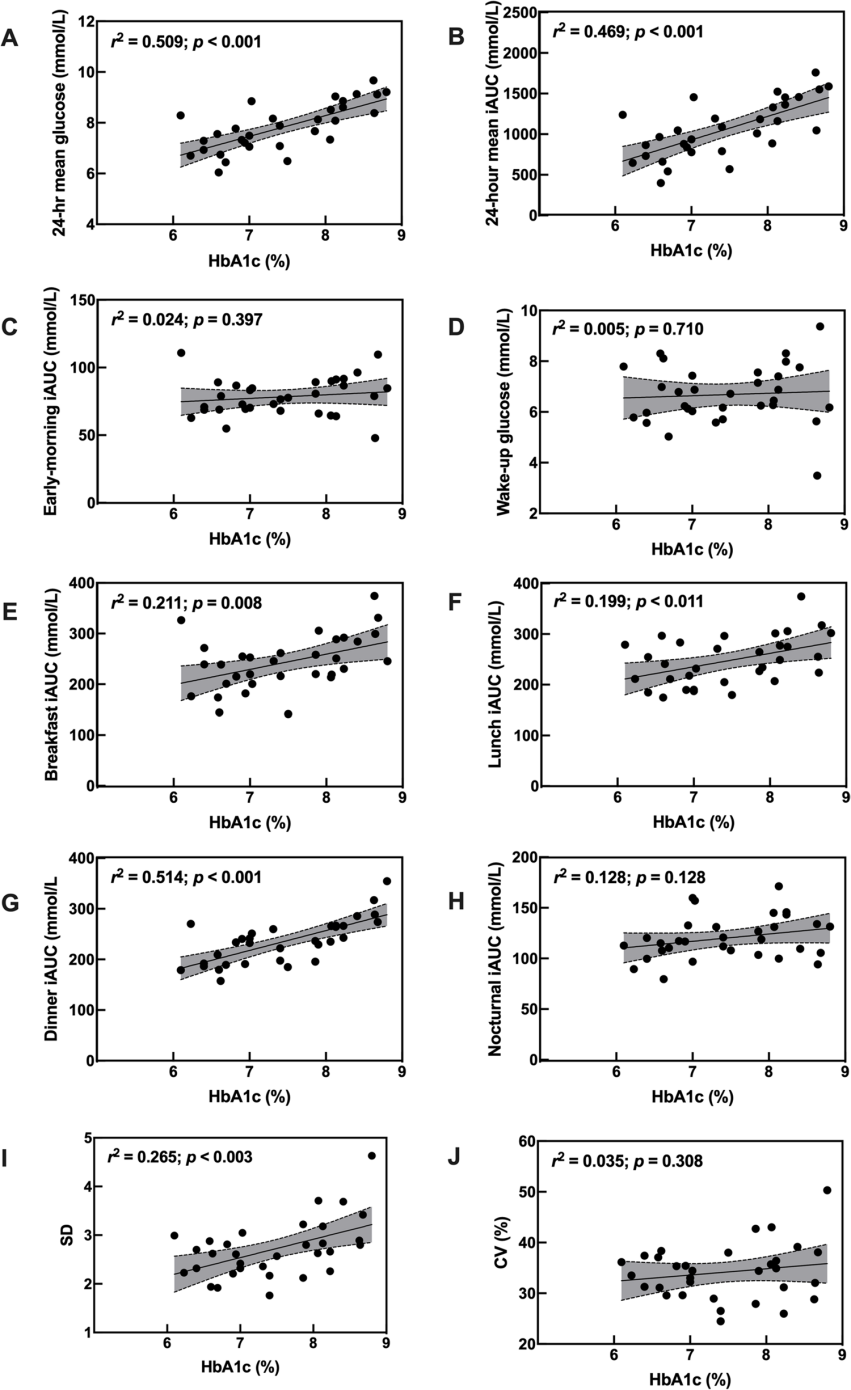
**A-J** Pairwise scatter plots of the interrelationships between CGM-derived metrics vs. HbA1c, with mean and 95%CI

Scatterplots of CGM-derived glycaemic metrics versus HbA1c measurements are shown in Fig. 3A-J. Notably, we observed statistically significant associations between postprandial glucose metrics (breakfast, lunch, and dinner iAUC) and HbA1c (*p* < 0.010), but not nocturnal (*p* = 0.128), early-morning glucose (*p* = 0.387), or wake-up glucose concentrations (*p* = 0.710). In addition, SD was significantly associated with HbA1c (*p* < 0.003), whereas CV was not (*p* = 0.308).

**Fig. 4:**
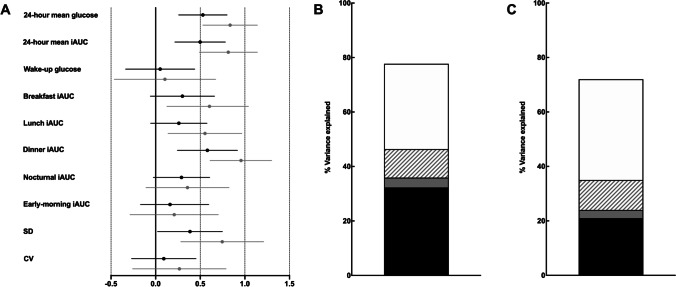
**A-C** (**A**) Mean (95%CI) difference in HbA1c (%) by 1-SD difference in CGM-derived glycaemic metrics. Estimated differences are unadjusted (grey) and adjusted (black) for age and BMI. (**B**) Proportion of variance explained in HbA1c by postprandial factors (black bar; breakfast iAUC, lunch iAUC, dinner iAUC), preprandial factors (grey bar; nocturnal iAUC, early-morning iAUC, wake-up glucose), glycaemic variability (hashed bar; SD, CV%), and non-glycaemic factors (white bar; age, BMI). (**C**) Sensitivity analysis of proportion of variance explained in HbA1c by postprandial factors (black bar; dinner iAUC), preprandial factors (grey bar; nocturnal iAUC), glycaemic variability (hashed bar; SD), and non-glycaemic factors (white bar; age, BMI)

Figure 4A shows the unadjusted and adjusted associations of HbA1c with CGM-derived glycaemic metrics. 24-h mean glucose, 24-h mean iAUC, and SD were strongly associated with HbA1c; these associations were robust following adjustment for confounders (age and BMI). In addition, postprandial metrics (breakfast iAUC, lunch iAUC, and dinner iAUC) were strongly associated with HbA1c. However, following adjustment for age and BMI, only dinner iAUC remained significant equating to a ~ 0.6%-point (~7 mmol/mol) increase in HbA1c per SD increase in dinner iAUC (Fig. 4A).

### Relative contributions of CGM-derived glycaemic metrics and non-glycaemic factors to variation in HbA1c

Figure 4B-C illustrates the proportion of variance in HbA1c explained by postprandial glucose, preprandial glucose, glycaemic variability, and non-glycaemic factors. In this cohort, 77% of the variance in HbA1c was explained by the included variables. Postprandial glucose accounted for a third of the variance explained (32%), compared to preprandial metrics which accounted for 4%, and glycaemic variability which accounted for 10%, of the variance explained (Fig. 4B). Further, the contribution of non-glycaemic factors was large (31% of the variance explained). Inclusion of only dinner iAUC, nocturnal iAUC, SD, as well as age and BMI, respectively, reduced the contribution from postprandial glucose (Fig. 4C).

## Discussion

Through the use of repeated CGM measurements over a sustained period of time under free-living conditions, we demonstrate that postprandial glucose exposure is a stronger determinant of HbA1c than preprandial glucose, nocturnal glucose, and glycaemic variability in people with T1D. Specifically, in our model which accounted for ~77% of the explained variance in HbA1c, we show that the evening-meal postprandial period is the single largest contributing factor to HbA1c.

Previous studies examining the exact role of postprandial glucose exposure and how it relates to HbA1c in people with diabetes are conflicting [18–22] with some studies showing a stronger relationship between postprandial glucose and HbA1c more than preprandial glucose [18, 23], and some studies showing the contrary [24, 25]. Most of these studies, however, have focused on people with type 2 diabetes (T2D) or mixed cohorts of T1D and T2D, and have assessed glucose exposure using data from a single day or a single-point SMBG value. In the present study, we used CGM to capture real-time glucose fluctuations allowing a comprehensive assessment of the individual contribution of discrete time intervals across the whole day, as well as glycaemic variability, on HbA1c in T1D patients.

Our finding that the evening-meal postprandial period, estimated as the 3-h iAUC for interstitial glucose following dinner, is the single largest contributor to HbA1c demonstrates the importance of tight mealtime glucose control in the clinical management of T1D and its associated complications. Large observational cohort studies demonstrate a clear and consistent association between postprandial hyperglycaemia and cardiovascular disease [26, 27] in people with T2D, although data assessing the precise relationship between postprandial glucose excursions and the development and progression of complications in T1D is lacking [28, 29]. However, considering that humans spend a large proportion of time in a postprandial state, it is logical that postprandial glucose control fundamentally influences HbA1c. In our cohort, an increase in each SD increment in dinner iAUC was associated with 0.6%-point (~7 mmol/mol) increase in HbA1c. A difference of this magnitude is clinically significant, representing ~5% increase in the long-term risk of a cardiovascular event [30]. It is important to note however, that individuals with an HbA1c of 6.9% (52 mmol/mol) or lower still have a 2-fold increased risk of cardiovascular disease compared to the general population [31] and therefore postprandial glucose control should remain a priority of self-management for all patients with T1D. In T2D, the %contribution of fasting or prandial glucose to HbA1c is at least in part influenced by baseline HbA1c levels; previously it has been shown that for those with baseline HbA1c of 7.3%, prandial glycaemia contributed to HbA1c by 70%, whereas those with a baseline HbA1c of >10%, fasting glucose contributed to HbA1c by 70% [32]. With average HbA1c levels ~7.5% in our patients, our findings complement prior findings in T2D, however further follow-up in patients with a wider range of glycaemic control will be necessary to determine whether the contribution of postprandial glycaemia to HbA1c is weakened in T1D.

Postprandial hyperglycaemia in T1D is multifactorial. Glucose responses to meals are impact by their timing, and nutrient quantity and composition, as well as challenges with accuracy in estimating total carbohydrate intake against insulin requirements [33–36]. Current guidance for mealtime self-management focuses predominantly on estimating carbohydrate type and amount to determine appropriate insulin dosing to maintain glucose levels within normal ranges [37]. Despite current practice, the use of meal carbohydrate content is a poor predictor of postprandial glucose responses [38]. Other methods which aim at estimating postprandial glucose responses include the glycaemic index, which quantifies the glucose responses to a single tested food type, postprandial glucose responses and its derived glycaemic load [39]. However, these methods have limited applicability in assessing PPGRs to meals consumed in real-life. This is because typical eating patterns consist of mixed-macronutrient meals of different food combinations and varying quantities [40] eaten at different times of day and influenced by the proximity of foods eaten previously. For example, our group [36], as well as others [41], have previously shown that aside from meal carbohydrate content, fat and protein content also influences postprandial handling and mealtime insulin requirements in T1D. In addition, postprandial glucose responses are not solely impacted by the intrinsic properties of food, but also by the personal physiological characteristics of an individual. Recently, Zeevi and colleagues [42] demonstrated a large degree of variability in the postprandial response to standardised test meals between individuals with and without prediabetes, a finding which we later replicated in individuals with T1D [43]. In our study, non-glycaemic factors, including age and BMI were the second largest contributor to explained variance in HbA1c which supports the notion that characteristics beyond food play an important role in postprandial glucose control. Presently, there is little international consensus for either the recommended measurement or specific targets for postprandial glucose levels in patients with T1D.

Glucose exposure during sleep, including early-morning, as well as wake-up glucose, and CV, were not associated with HbA1c in this study. 91% of our patients encountered hypoglycaemia during sleep, with the average time spent in hypoglycaemic ranges between ~30 90-min. Previously, it has been reported that a 1% increase in HbA1c is associated with 41% decrease in the risk of nocturnal hypoglycaemia [44]. Further, we observed a noticeable rise in glucose levels during the early hours of the morning in ~20% of our patients (Fig. 1D). A transient increase in morning time glucose concentrations, termed the dawn phenomenon, is a well-established and frequent event in T1D [45]. However, on average, wake-up glucose concentrations were largely comparable to mean 24-h glucose levels, and lower than postprandial glucose concentrations. In our analysis, both early-morning glycaemia (captured 2-h prior to wake-up) as well as wake-up glucose levels were not significantly associated with HbA1c indicating that the dawn phenomenon played a relatively minor role in influencing long-term glucose control in our subjects. Generally, stable glucose levels are defined as a CV <36% [46]. The average CV in our cohort was ~34%, although this ranged considerably between patients (Range: 25–50%), and mean 24-h glucose SD was 2.7 mmol/L which is consistent with previous assessments of glycaemic variability in T1D [44]. It possible, that heterogeneity within our sample contributed to a lack of association between these metrics and HbA1c.

A limitation of this study is its observational nature as the two RCTs from which data was reanalysed were not initially designed to assess the association between CGM-derived metrics and HbA1c. In addition, our study sample consisted of males in relatively good diabetes control from a single centre, which hampers the generalisability of results. Considering that the duration of diabetes, type of treatments, sex, ethnicity, and nutritional status are likely to have an important effect on HbA1c, further studies in a broader, more heterogeneous population of T1D individuals is needed to determine whether these factors as well as other may explain residual variance. Another important consideration is that HbA1c is modulated by intracellular glucose levels and that glucose uptake and erythrocyte lifespan is inter-individual [47, 48]. As such we cannot categorically rule out a role for these erythrocyte-related variables and their potential mediating impact on our study findings, Recently, our group has proposed a model which, incorporating erythrocyte lifespan, attempts to address limitations in laboratory HbA1c [49–52]. However, the method used provides only an approximate measure and therefore the interaction between fluctuations in daily glucose levels and erythrocyte parameters remains an area for future work.

The findings of this analysis show that postprandial glucose, specifically evening-time postprandial glycaemia, is a significant contributing factor to HbA1c in patients with T1D. These data highlight the importance of tight mealtime glucose control in the clinical management of T1D and its associated complications and suggest evening-meal glucose should feature as a key treatment target.

## Data Availability

The data that support the findings of this study are available on request from the corresponding author.
